# Digital media technology in drama education: educational value and mental health empowerment from a symbolic interactionism perspective

**DOI:** 10.3389/fpsyg.2026.1810918

**Published:** 2026-04-22

**Authors:** Peng Wei, Majid Murad

**Affiliations:** 1 School of Culture and Art, Xinjiang Arts University, Urumqi, China; 2 School of Innovation & Entrepreneurship, Guangzhou University, Guangzhou, China

**Keywords:** China, digital media technology, drama education, educational value, mental health, symbolic interaction theory

## Abstract

**Background:**

Digital media technology offers diverse educational methods, shaping interaction and communication within the educational process and further embodying profound value in promoting students' mental health. Drawing on symbolic interactionism, this study systematically analyzes the educational value and mental health empowerment pathways of digital media technology in drama education. It re-examines the role of digital media technology in drama education and clarifies the direction for leveraging such technology to develop drama education.

**Methods:**

The research employs an ethnographic methodology, conducting in-depth personal interviews with 30 drama education professors in China. Drawing on the four dimensions of symbolic interactionism—“subject, content, behavior, situation”—the study analyzes the primary educational value of digital media technology in university-level drama education and clarifies the internal logic connecting technology, interaction, and psychological development.

**Results:**

The study finds that digital media technology, represented by virtual reality, is being used with diminishing frequency, and the role of educators is shifting from “performance instructor” to “technology guide.” Simultaneously, digital media technology transforms dry educational content into “vivid and immersive” virtual-real scenarios, providing teachers and students in drama education with “authentic” and immersive digital educational content, along with distinctive emotional experiences. Digital media-empowered drama education effectively enhances students' psychological resilience and social adaptability, offering a feasible pathway to achieve the dual objectives of “teaching quality enhancement” and “psychological empowerment” in education for the new era.

**Conclusion:**

The changes in content, action, and environment brought about by digital media technology in drama education reflect the requirements of the integrated development of art and technology, as well as new opportunities and challenges for drama education. Digital media technology builds a crucial bridge for the deep integration of symbolic interactionism with drama education and mental health. It also provides theoretical reference and practical insights for the digital transformation of drama education and the organic integration of mental health education.

## 1 Introduction

The term “digital” derives from the Latin word digitus, meaning “finger,” as fingers historically served as primary tools for counting and representation (Lee, 2023). Digital media refers to the collection, processing, and dissemination of text, sound, and images through digital means. Digital media technology is an information technology for the dissemination, storage, and management of various digital media, such as photos, audio, video, and text, through computers, the Internet, and wearable devices (Antonietti et al., 2022). In the digital era, digital media technology has achieved significant advancements across social, cultural, economic, and artistic domains. Innovations such as virtual reality (VR), holography, and the metaverse are widely used in concerts, exhibitions, and museums, revolutionizing industry practices and expanding human sensory engagement (Jeong, 2020). As a transformative medium, digital technology not only reshapes education but also emerges as a critical conduit for global interaction, aligning with societal needs and human progress.

Drama education, a cornerstone of artistic pedagogy, is profoundly significant in fostering creativity, emotional intelligence, and social collaboration. Through embodied practices, drama enables students to reconstruct their perceived world, reclaim their agency as sensory beings, and creatively shape their lives (Xiao, 2024). Recognized as an effective interdisciplinary tool, drama education enhances core competencies such as imagination, moral reasoning, and physical coordination, while cultivating self-expression and personality development (Choi and Oh, 2020). Despite the revitalizing impact of digital media technology on drama education, its educational value remains underexplored and fragmented. From the perspective of symbolic interaction theory, the core value of technology lies in reconstructing symbolic meanings and innovating interactive scenarios to deepen educational significance. Current practices in higher education often reduce technology to a superficial tool, failing to translate its potential into pedagogical efficacy. Teachers exhibit ambiguous understandings of technology's educational functions, leading to directionless integration in curriculum design, while students' overreliance on technological tools risks undermining artistic autonomy. This disconnect between technological applications and educational objectives necessitates a re-examination of the intrinsic relationship between digital media technology and drama education through a value-oriented lens.

The core characteristics of digital media technology are manifested as immersion, interactivity, and data-driven capabilities. Virtual reality breaks through the limitations of physical theaters through multi-dimensional spatial construction, while artificial intelligence can capture performance details in real time and provide dynamic feedback. However, the superficial application of technology has revealed deeper contradictions: technological intervention has yet to harmonize with the humanistic core of drama education. The central question this study addresses is: what educational value does digital media technology hold in drama education? Through in-depth interviews with professors in university drama departments and the application of symbolic interaction theory, this research analyzes the educational value of digital media technology in higher education drama programs. It aims to clarify the direction of digital media technology in university-level drama education, providing guidance for more effective utilization of such technology in drama education practices.

Additionally, this study explores digital media technology in drama education from the perspective of mental health. Drama therapy, as a form of group psychotherapy and expressive arts therapy, originated from psychodrama proposed by Jacob Moreno in the 1920s, with its framework established in the United Kingdom and the United States in the 1970s (Emunah and Williams, 1996). Drama therapy is an active and experiential psychotherapy modality that intentionally and systematically uses drama/theater processes as a primary means to achieve psychological growth and change within a psychotherapeutic relationship (Feniger-Schaal and Orkibi, 2020). Over the years, drama therapy has gradually evolved into one of the most popular forms of art-based healing. In drama therapy, participants take on the roles of playwrights, actors, and audiences, achieving psychological transformation through the power of improvisation (Johnson, 1991). Currently, the digital transformation of drama education is not only an inevitable choice driven by technological innovation but also a contemporary extension of the essence of arts education. Its core value lies in revitalizing the vitality of traditional theater by facilitating cross-temporal and spatial dialogues with classical texts through technological media, promoting educational equity by enabling high-quality drama resources to transcend geographical limitations, and enhancing students' core competencies, including creativity, social collaboration skills, and emotional management.

## 2 Theoretical background

### 2.1 Literature review

In this society, technology is not a specific factor or one of many dimensions, but the precondition of all reality and actualization (Kellner and Marcuse, 2013). The impact of technology on individuals, society, and nature is always a coexistence of positives and negatives. Under such complexity, understanding what technology brings to humanity is more urgent and meaningful than understanding the technology itself (Shen, 2015). Therefore, this study begins by reviewing the literature on the values and classifications of digital media technology, its artistic and educational value, particularly in higher education. Building on this foundation, the paper examines current research on the educational value of digital media technology in drama education within universities.

#### 2.1.1 Educational value

Value orientation refers to the process through which a value subject directs activities toward value objectives during value-related practices, reflecting the overall trend and developmental direction of the subject's value concepts (Ruan, 2010). As a key concept in value philosophy, value orientation embodies the manifestation of values in social contexts. It represents the stance, attitude, and fundamental inclinations demonstrated by a subject in value activities, rooted in cognitive processes and ultimately guiding behavioral practices (Huang, 2022). Educational value orientation, in turn, refers to the value relationships that educational subjects (e.g., educators and institutions) follow, pursue, construct, and realize in practical educational activities (Tao, 2005). Based on these definitions of value orientation and educational value orientation, this study further explores the conceptual boundaries of “educational value.”

The term “educational value” was first proposed by the 19th-century British educator Herbert Spencer (Zhang, 2020). Chinese academia holds diverse perspectives on its connotation. Some scholars define educational value as the significance or role of education for individuals and society, categorizing it into political, economic, social, cultural, and pedagogical values, depending on the educational subject (Zhang, 1999). Others classify it into instrumental vs. intrinsic values, ideal vs. practical values, and product vs. process values (Bernstein, 2001). Another view posits that educational value addresses the question of “what is the purpose of education,” aiming to clarify its significance for subjects (Zhang, 2022). Despite ongoing debates, three primary perspectives emerge:

*Educational value as the satisfaction of subject needs:* this perspective defines educational value as the extent to which education, as a social system, satisfies the developmental needs of societal and individual subjects (Wang, 1996). Critics argue that while this view emphasizes educational goals, it overlooks the value of educational processes and interactions among educational elements.

*Educational value as a relational construct:* Wang (2006) conceptualizes educational value as a specific relationship between the attributes of educational phenomena (as objects) and the needs of human subjects in social practices. Similarly, Mingers (1994) views it as the fulfillment of educational demands through interactions between subjects and objects. While this relational approach highlights interdependence, it risks oversimplifying the dynamic impacts of education.

*Educational value as individual and societal fulfillment:* Nucci (1982) distinguishes between societal development and individual development dimensions of educational value. Hu (1997) further associates it with how educational characteristics, functions, and outcomes align with human needs. However, such frameworks may neglect the multidimensional linkages between subjects and objects. In addition, South Korean scholar Kim (2018) explored the theoretical and practical implications of educational values from the perspective of teachers, reexamining Dewey's concepts of “continuity” and “interaction” as principles of educational value and educational experience. By integrating these concepts with teachers' pedagogical practices, Kim sought to understand the formation, intellectualization, and free expansion of educational values, discussing educational value in terms of both “teaching process” and “learning outcomes.”

Korsgaard (1983) identified three major categories of value addressed by moral philosophers: the rightness or justice of actions, policies, and institutions; the goodness of objects, ends, lives, etc.; and the moral worth or moral goodness of character, dispositions, or actions. Gatley (2021) put forward two propositions in defining educational value: the first is that something is only educationally valuable if it is good for the person being educated. The second is that ascribing educational value to an activity, state of affairs, or good confers some claim to consideration for its inclusion in the curriculum. In discussions of the value of art education, some scholars argue that art's educational function lies in its capacity to stimulate the imagination, thereby creating, shaping, and forming experience. Art is a process in which the self-confronts its subjective inner world and molds it into objects that can be acquired within the unique cultural world of human beings (Bayer, 2006).

Synthesizing these perspectives, this study defines educational value as the mutual adaptation and interaction between educational subjects (e.g., learners) and objects (e.g., knowledge), dynamically generated through educational activities. To analyze educational value, it is essential to situate it within educational processes and outcomes. Moreover, educational value orientation should strike a balance between societal and individual priorities (Hu, 2020).

#### 2.1.2 The value of digital media technology in art

Digital media's application across domains serves as a novel tool, enabling artists to innovate existing paradigms and techniques (Marner and Örtegren, 2013). With advancements in computer multimedia and wireless internet technologies, digital media art has emerged as a groundbreaking form that merges rational and rational thinking through digital platforms and modern media (Zhao and Wang, 2020). As economies and societies evolve, digital media technology has been widely adopted in artistic practices, demonstrating profound value.

The influence of digital technology on the field of arts and culture is significant and diverse (Mardiani et al., 2024). From the literature on the impact and role of digital media technology in art, it can be seen that digital media technology not only endows art with external values such as tool value, but also provides new vitality for art creation and the intrinsic value of art itself. Emerging technologies are not only the wings of artistic creation but also catalysts for its evolution. Whether digital media, VR/AR, or even AI, they are reshaping people's lifestyles and ways of thinking to varying degrees, bringing about a profound innovation in artistic creation (Li and Su, 2025). Integrating art and technology has become a significant force in the creative world, presenting opportunities and challenges for artists, creatives, and consumers (Strandgaard Pedersen et al., 2019).

In the theater, scholars have explored the role of digital media technology. Barber (2016) argues that digital media technology enriches theatrical narratives through nonlinear storytelling, redefines stage aesthetics, and enhances audience empathy. Dergunova and Popova (2023) observe that digital tools transition from mere mediators to partners, reconfiguring relationships between objects, tools, and subjects. In participatory theater, digital technology functions as a dynamic “actor,” co-constructing experiences with human and non-human participants (Burnett et al., 2018). These studies underscore digital media technology's dual role as both tool and co-creator in theatrical art. Theater, as a medium of communication and education, holds significance for both artistic and pedagogical development. While scholars increasingly classify digital media's value in art, rapid technological advancements often outpace research. Factors influencing its value in art remain underexplored, necessitating further inquiry. Nonetheless, digital media technology harbors immense potential to drive the prosperity of theater and other art forms.

#### 2.1.3 The value of digital media technology in education

The proliferation of digital media technology in education heralds an era of disruption and opportunity. Educational environments and stakeholders continuously adapt to evolving challenges, including curricular innovation, cognitive shifts, and technological integration (Kenwright, 2020). Digital media technology revolutionizes pedagogy, enriches content, enhances outcomes, and fosters educational socialization. Current research focuses on three key areas:

*Instrumental value:* digital media technology revitalizes traditional teaching methods. For instance, multimedia displays, online platforms, and virtual simulations rejuvenate calligraphy education (Huang and Qiao, 2024). Web-based social technologies enable the creation of innovative informal learning environments (Kivunja, 2015). As applications expand, digital media's value in classroom demonstrations, assessments, and tutoring continues to evolve.

*Interactive value:* digital technology reshapes undergraduate education by transforming student learning experiences (Henderson et al., 2015). In ideological education, digital tools enhance awareness, communication, and social skills while reducing costs and stress (Xu et al., 2024). Generative AI and interactive media foster student engagement, personalized inquiry, and collaborative learning.

*Societal value:* educational technology accelerates modernization and equity by bridging urban–rural and interregional resource gaps (Wang et al., 2023). The integration of digital media in drama education remains underexplored, yet its potential to enhance cultural preservation and innovation is evident. For example, the metaverse offers immersive methods for heritage conservation and global accessibility (Alivizatou-Barakou et al., 2017). AI also democratizes learning for marginalized groups, including refugees and people with disabilities (Pedro et al., 2019). International research on the educational value of digital media technology provides critical insights. While its application significantly improves pedagogy and equity, theoretical studies lag behind technological advancements. Future efforts should prioritize empirical research, theoretical depth, and interdisciplinary collaboration to optimize digital media's role in education.

*Emotional value:* in the era of human–machine symbiosis, intelligent technologies have been widely integrated into educational contexts, leading to profound changes in how teachers and students interact and in their emotional connections (Wu et al., 2025). A systematic review by Kkeli and Jimoyiannis (2025) of 33 studies from 2012 to 2022 shows that digital narrative interventions can effectively promote learners' emotion recognition and expression, emotional engagement and regulation, and social awareness and interpersonal skills. The empowering form of narrative enables learners to organize scattered emotional experiences into shareable, reflective stories, where technology serves not as a mediator that replaces emotions but as a “scaffolding” for the externalization and reconstruction of emotions. A systematic review by Mukhemar et al. (2025) shifts the focus from students to teachers, exploring the application of technology-enabled social and emotional learning (SEL) in teacher professional development. The study finds that digital tools such as online platforms, virtual simulations, and mobile applications have been used to cultivate emotion regulation, self-awareness, and cross-cultural adaptability among university teachers. This perspective shift holds significant implications: technology is not only a tool for emotional education but also a medium for cultivating the subjects of emotional education.

### 2.2 Symbolic interactionism

Symbolic interaction theory has developed in the light of theorists such as Dewey, Cooley, Parks, Mead, etc. (Aksan et al., 2009). Symbolic interactionism refers to the process by which individuals engage in dialogue and communication through symbols within specific contexts. Its interpretation can be framed in terms of the interactive relationships among three elements: symbolic features, construction processes, and cognitive processes. Symbolic interactionism is a micro-level theoretical framework and perspective in sociology that addresses how society is created and maintained through repeated interactions among individuals Carter and Fuller, (2015). With the evolution of symbolic interactionism, scholars like Turner have sought to integrate it with other theoretical frameworks to address complex social dynamics. For instance, (Downer et al. 2015) employs symbolic interactionism as an analytical framework to theorize teacher–student interactions in smart classrooms across four dimensions: subject, content, behavior, and situation. The subject encompasses teachers and students; the content includes cognitive and affective elements of interactions; the behavior involves verbal and non-verbal actions; and the situation refers to the learning environment, including technological infrastructure and pedagogical atmosphere.

#### 2.2.1 Subject

In symbolic interactionism, the “Subject” denotes an individual's identity and self-concept. In drama education, the subjects are teachers and students. The theory posits that the self emerges from the internalization and subjective interpretation of social realities, shaped through social interactions. Self-development is a gradual process that requires a student-centered approach in education. Symbols, classroom contexts, and interactive feedback are critical to student learning (Chen, 2024).

#### 2.2.2 Content

In symbolic interactionism, the element of “Content” primarily focuses on the meanings, information, and symbols conveyed during social interactions. In drama education, teachers and students interact through verbal and non-verbal symbols in the teaching process, ultimately achieving a shared understanding of the content within a unified communication system. This includes both cognitive alignment and emotional resonance (Huan, 2023). In this study, “content” mainly refers to the substance of drama education, specifically comprising two aspects: the educational content itself and the medium through which it is delivered.

#### 2.2.3 Behavior

In symbolic interactionism, the element of “behavior” focuses on the specific behaviors of individuals within social interactions (Van de Pol et al., 2010). Drama education is a process of interaction mediated by symbols, which primarily includes teacher–subject/object interaction, student–subject/object interaction, teacher–student interaction, and student-student interaction. These interactions further encompass both verbal and non-verbal symbolic exchanges. Within these interactions, teachers and students use symbols to share meanings, develop self-awareness, and continually adjust and refine their understanding through social engagement. Thus, the value of digital media technology in such interactions lies in its ability to effectively construct drama education experiences, facilitate the achievement of educational goals, and advance drama education.

#### 2.2.4 Situation

In symbolic interactionism, the element of “situation” refers to the contextual conditions in which social interactions occur, encompassing aspects such as the physical environment, socio-cultural setting, and technological milieu. In the context of drama education, it primarily denotes the learning environment. Symbolic interactionism posits that the development of mind and self is a process of socialization, which similarly applies to the growth and formation of individual students. The concept of learning environment is broad and complex, with varying definitions depending on the research context and objectives. As a significant component of the material environment in contemporary learning settings, digital media technology is of considerable importance to drama education. Given that the learning environment is a multidimensional construct, this study analyzes the development of digital media technology in the context of the “situation,” primarily from the perspective of the physical environment in drama education. This includes two dimensions: the digital media technology environment within the classroom and outside the classroom.

## 3 Methods

This study is grounded in the “humanistic” nature of educational activities and employs symbolic interactionism as its theoretical framework to investigate the interplay between digital media technologies and drama education. Adopting a qualitative research paradigm, the research utilizes ethnography to explore the dynamic and complex educational phenomena. Through a postmodern lens, the ethnographic approach emphasizes interpreting participants' educational practices and value cognition within specific sociocultural contexts. Purposive sampling was conducted to select 30 authoritative experts in Chinese higher education drama pedagogy. Participants were professors from institutions ranked in the Fourth Round of China's Discipline Evaluation for Drama and Film Studies, all with at least 3 years of experience in integrating digital media technologies (including VR) into drama education and active engagement in curriculum design within the past 3 years.

The study employed semi-structured interviews, conducted over 3 months in a hybrid (online and offline) format, with each session lasting 60–90 min. The research design was based on three considerations: first, practical knowledge is embodied in professional experiences, and in-depth interviews effectively uncover tacit knowledge. Second, symbolic interaction theory necessitates attention to the multidimensional interactions of “subject-content-behavior-situation.” Third, ethnography enables the capture of cultural codes within educational settings through prolonged immersion.

To design the interview questions, a semi-structured interview protocol was developed based on the theoretical framework of symbolic interactionism, comprising four dimensions: self, content, behavior, and environment. In the self-dimension, the focus is on how digital media technology influences the role perceptions and identity construction of teachers and students, as well as the associated confidence, sense of achievement, and psychological growth that result from its application. The content dimension examines how technology enriches the forms of expression in drama education content and how changes in content presentation affect students' emotional resonance and psychological identification. The behavior dimension explores how technology optimizes patterns of teacher–student interaction and peer interaction, with particular attention to its role in enhancing students' willingness to express themselves, psychological safety, and positive emotional support. The environment dimension investigates the impact of the learning environment constructed by digital technology on students' sense of psychological belonging, participation thresholds, and emotional experiences, with a focus on how the technological environment alleviates creative pressure and empowers students' mental health.

The interviews were conducted in three stages: pre-interview, formal interview, and post-interview. The pre-interview was primarily used to gather background information and to inform participants about the interview procedures. The post-interview served as a supplementary session, conducted sometime after the formal interview in the form of a narrative interview, allowing for a more comprehensive understanding of the participants. During the interviews, with the participants' consent, the entire process was audio- and video-recorded, supplemented by field notes to ensure that the collected interview data were comprehensive, accurate, and in-depth. After the interviews, the data were organized into systematic interview reports, which were then shared with the participating professors for verification, accuracy checking, and to solicit additional input or suggestions for revision.

In the data analysis phase, coding categories were developed through a review of the literature and relevant case studies. Interview notes were classified and analyzed to identify common themes and patterns, which were subsequently coded. Through repeated cross-checking between the interview records and the coding categories, revisions were made to ensure consistency. The coding process was guided by the four dimensions of symbolic interactionism—self, content, behavior, and environment—under which the interview content was further categorized, and each category was interpreted in terms of its specific meaning.

Taking the subject dimension as an example, in the open coding stage, initial concepts and labels were extracted line by line from the original interview transcripts, such as “technology provides more platforms and opportunities,” “technology benefits humanity,” “reduced role of teachers in the classroom,” “shift from performance instructor to technology instructor,” “students need to enhance digital competence,” “technology enables immersive emotional expression,” and “lowered psychological barriers.” In the axial coding stage, these open codes were grouped and integrated: codes related to the teacher's role were synthesized into the theme of “transformation of the professor's role,” while codes concerning students' abilities and emotional experiences were synthesized into the themes of “changes in students' self-perception” and “emotional support,” thereby forming higher-order thematic categories. In the selective coding stage, centered on the core category of “the educational value of digital media technology for the self,” themes such as “transformation of the professor's role,” “changes in students' self-perception,” and “emotional support” were integrated into a unified explanatory framework. This ultimately led to the core conclusion that “digital media technology realizes its educational value for the self in drama education by reshaping the roles and emotional experiences of both teachers and students.”

Ethical considerations included anonymizing participants (coded as A1, A2, A3…) and safeguarding their privacy. All referenced materials and literature were cited objectively, while personal information was omitted to ensure participants' security and their willingness to engage in candid, in-depth discussions. The study's findings are evidence-based, with arguments rigorously substantiated to uphold academic integrity and methodological rigor.

## 4 Findings

### 4.1 Educational value of digital media technology on the “subject” and its emotional support for student mental health

In drama education, both educators and students serve as primary agents who utilize digital media technology. Interpreting the pedagogical value of these technologies from their perspectives—analyzing their significance and aspirational goals—enables stakeholders to harness digital media tools more effectively, thereby advancing the overarching objectives of drama education. When discussing the educational value of digital media technology for the “subject” drama professors unanimously affirmed its contributions to pedagogy. While acknowledging the existence of “technological nihilism” in broader societal discourse, which overemphasizes technology's adverse effects in education, participants emphasized that the benefits of technology remain dominant despite occasional challenges.

“*As a product of societal progress, digital media technology drives advancements for individuals and humanity as a whole. It embodies human ingenuity and will inevitably reciprocate by enriching human development across all domains, including education.” (A4-PII)*

#### 4.1.1 Transformation of professors' roles

Digital media technology has catalyzed shifts in educators' roles. As these technologies permeate drama education, instructors' traditional responsibilities are being reconfigured. First, professors' “stage presence” in classrooms has diminished. Digital tools now mediate content delivery and interactive modes, diversifying students' access to knowledge. Participants noted that while technology assumes some conventional teaching tasks—such as delivering foundational drama knowledge—the essence of drama education, rooted in emotional expression and transmission, remains irreplaceable. Second, educators are transitioning from “performance mentors” to “technology facilitators.” Traditional drama pedagogy prioritized scriptwriting, acting techniques, and creative processes, with limited integration of digital tools. However, the proliferation of technologies like virtual characters and digitized stage design necessitates that professors provide technical guidance alongside artistic instruction.

“*With the rise of digital media, students increasingly require technical support in areas such as virtual reality integration—skills previously absent from drama curricula. Today, educators must first master emerging technologies themselves before guiding students in their application.” (A7-PII)*

#### 4.1.2 Changes in student self-perception and emotional support

Digital media technology has brought about significant changes in students' self-perception. Professors of drama education generally agree that advancements in digital media technology demand higher levels of digital literacy from students. In drama creation, students are required to deepen their understanding, awareness, and application of these technologies. With the increasing integration of digital media technology in drama education classrooms, students face new challenges. The development of artificial intelligence has diversified classroom roles, broadened students' access to knowledge, enriched the presentation of teaching content through technologies such as VR, and made the use of smart devices in classroom interactions increasingly common. This necessitates that students enhance their innovative capabilities, improve their digital skills through coursework, and use digital media technology to create and disseminate drama.

Simultaneously, the integration of digital media technology provides new pathways for drama creation and expression, while also offering new support and possibilities for students' emotional development and psychological wellbeing. Emerging technologies such as VR provide drama education students with opportunities for immersive emotional expression and self-exploration. The development of these new technologies in drama education helps alleviate students' creative pressures and enhances their confidence and sense of accomplishment in drama learning.

“*Digital media technology introduces new ways for students to experience and express emotions. Moving from traditional text-based and physical expression to emotion simulation and release through VR scenarios and interactive narratives, drama education has become more therapeutic and inspiring.” (A14-PII)*

Drama inherently possesses functions of emotional education and psychological healing, and the integration of digital media technology further expands this dimension. Through technological means such as VR scenario reconstruction, students can receive immediate emotional feedback and positive psychological reinforcement in artistic practice, thereby accumulating positive emotional experiences and enhancing self-efficacy. Additionally, digital media technology helps students better understand and manage their emotions, enhances empathy and psychological resilience, and makes the drama education process more enjoyable and meaningful.

“*Digital media technology opens a new window for drama education in higher education institutions. It serves not only as a teaching tool but also as emotional support for drama education students. The use of digital tools lowers the psychological barriers to participation in drama, particularly helping students with introverted, anxious, or socially avoidant tendencies gradually build expressive confidence and a sense of belonging. It enables more students to willingly and effectively express themselves through drama, which holds profound significance for mental health.” (A8-PII)*

### 4.2 Educational value of digital media technology on “content” and its positive adjustment for student mental health

#### 4.2.1 The educational value of digital media technology for “content”

As a product of societal development, digital media technology embodies human ingenuity and serves as a primary non-verbal symbol in drama education. It facilitates cognitive and affective alignment between teachers and students regarding educational content. Scholars note that students exhibit heightened curiosity toward new digitalized drama content formats, which enhance engagement compared to traditional methods. Digital content offers heightened realism, immediacy, and immersion. The shift from text- and image-based content delivery to immersive formats (e.g., virtual characters, VR videos) provides students with novel learning experiences. Beyond accessing vast knowledge repositories, students receive personalized content through big data analytics platforms. Furthermore, digital media technology has revolutionized the creation and presentation of theatrical works. Technology now plays an integral role in drama production, transitioning from a human-centric focus to a “human-technology synergy.” Interviews with university professors reveal that the educational value of digital media in drama content lies in two dimensions: introducing new content and transforming existing content.

Scholars assert that digital media technology represents a cognitive breakthrough and a tool for exploring uncharted domains. Its embedded value systems and influence on thinking patterns are vital for aligning drama education with societal progress. Students must acquire skills to use digital media to understand, create, disseminate, and advance drama.

“*The rise of digital theater has led creators to integrate VR, AR, and 3D animation into productions, expanding content and audience appeal. Digital theater now constitutes a core component of drama education and a key research focus.” (A5-PII)*

“*The fusion of technology and art drives contemporary drama development, making tech-integrated content indispensable in curricula.” (A28-PII)*

Digital media technology not only constitutes part of drama education content but also reshapes its traditional pedagogical frameworks. Scholars argue that digital media technology revolutionizes drama education content across multiple dimensions, from artistic creation to curricular structures. It facilitates both the preservation and innovative evolution of educational content while introducing novel emotional experiences. By diversifying content formats and enhancing accessibility, digital media fosters unified understanding between teachers and students, thereby improving pedagogical efficacy.

“*Digital media elevates the political, economic, and cultural value of drama education, balancing its inherently emotional nature with technology-driven warmth. This synergy stimulates creativity and enriches content inheritance.”* (A30-PII)

#### 4.2.2 The positive adjustment of digital media technology for the mental health of drama education students

Professors of drama education believe that integrating and reconstructing drama education content through digital media technology not only drives changes in educational content but also profoundly facilitates positive adjustments in students' mental health. Traditional drama education content is constrained by physical stages, paper-based scripts, and the requirement of spatial-temporal synchronicity, with emotional transmission often relying on real-time interactions and empathetic imagination. In contrast, digital media technology transforms drama content from static text into dynamic, interactive “living” narrative fields through digital script interactivity, virtual scene generation, and emotional character modeling. This “activation” of content transforms drama into an effective medium for students to identify, express, and regulate their emotions.

Simultaneously, tools such as digital feedback systems significantly lower the barriers to entry and psychological risks associated with drama participation. Students can anonymously experiment with different roles in virtual spaces, repeatedly refine their performances through digital editing, and gradually accumulate successful experiences. They can also receive personalized guidance from intelligent tutoring systems, reducing anxiety related to external evaluations. This process of empowerment enables students to build expressive confidence and social competence in a safe digital environment, particularly helping to alleviate “stage fright” and creative anxiety.

*With the intervention of digital media technology, drama education has shifted from “a showcase for the skilled few” to “an expressive medium accessible to the many,” achieving dual enhancements in psychological inclusivity and competency recognition at the digital level (A19-PII)*.

*Digital media technology provides students with more space, allowing them to choose content based on their emotional state, which helps them better express and regulate themselves. In some cases, it even brings students a sense of stability, as if they have gained a new source of support. (A8-PII)*

### 4.3 Educational value of digital media technology on “behavior” and its collaborative enhancement for student mental health

Drama educators emphasize that digital media technology endows pedagogical interactions with three defining characteristics in drama education: visualization, intelligence, and interactivity. Unlike traditional methods, digital visualization offers immersive, scenario-based experiences, enabling students to engage with theatrical content as if they were physically present. Intelligent interactions involve digital tools assuming active “roles”—interpreting user commands to deliver precise information. Interactivity prioritizes efficiency and feedback, particularly through human–machine collaboration, which fosters novel behavioral patterns in theatrical creation.

#### 4.3.1 Teacher–digital interaction: opportunities and challenges

Digital media empowers drama educators to rapidly access pedagogical resources, diversify instructional formats, and optimize workflow efficiency. While initial adaptation to new technologies may pose challenges, sustained engagement allows teachers to master tools such as VR and AI-driven platforms, ultimately enhancing scenario design and experiential learning.

“*Early struggles with digital tools are outweighed by their efficiency, versatility, and capacity to elevate teaching experiences.” (A16-PII)*

“*Educators must actively embrace digital media to remain competitive and innovative in the digital era.” (A26-PII)*

Educators are urged to leverage technology for personalized guidance, skill development, and professional growth. By cultivating digital fluency, teachers transform technology from a mere tool into a catalyst for pedagogical innovation.

#### 4.3.2 Student–digital interaction: empowerment and caution

In comparison to the teacher's interaction with digital media technology, the drama education professors identified many favorable aspects and some concerns. They believe that students use digital media technologies to engage in independent learning, complete assignments, and take tests. Digital media technologies broaden students' access to teaching resources and offer diverse experiences. Students like new things and emphasize emotional experience, and these digital media technologies satisfy their needs well.

“*The process of students interacting with digital media technologies is also the process of continuously improving their creative thinking. The new digital media technology is highly significant in enhancing students' innovation and creativity. Students in theater education need not only artistic connotation but also technological thinking.” (A12-PII)*

Theater education professors believe that digital media technology presents opportunities and challenges for theater education students. Drama education students need to learn to make effective use of digital media technology so that it can be an aid to learning. But at the same time, they should not rely too much on digital media technology and should think more actively for themselves. In interactions with digital media technology, students need to improve their judgment and overall ability to use it.

#### 4.3.3 The value of digital media technology in teacher–student interaction

Digital media technology extends teacher–student interaction in drama education beyond the classroom, offering novel modes of engagement that transcend spatial boundaries, enhance efficiency, and enrich experiential quality. Educators emphasize that digital tools fundamentally reshape pedagogical dynamics, fostering deeper connections and more effective communication.

“*Speed and Precision: Digital platforms enable instant communication, allowing instructors to distribute materials, assign tasks, review assignments, and analyze learning data in real-time. This immediacy ensures teachers can accurately monitor student progress and tailor interventions.” (A7-PII)*

“*Immersive Engagement: Transitioning from text-based instruction to multimedia formats (e.g., videos, VR) transforms how students perceive theatrical content. Previously reliant on abstract interpretation, learners now experience immersive, lifelike representations of dramatic works, deepening their understanding.” (A9-PII)*

In practice, educators leverage digital tools to present, disseminate, reflect on, and refine curricular content. Online discussions, interactive teaching software, and digital assessment systems have become integral to modern drama courses. Universities recognize these technologies as catalysts for pedagogical innovation, urging educators and students to iteratively refine their practices to maximize educational value.

#### 4.3.4 The value of digital media technology in student-to-student interaction

Digital media technology revolutionizes peer interaction by diversifying formats, improving efficiency, and fostering democratic participation. It dissolves physical barriers, enabling cross-boundary collaboration and infusing interactions with socially relevant content that stimulates creative thinking.

“*Diverse Modalities: Students embrace novel interaction paradigms facilitated by big data and AI, boldly expressing ideas and emotions while receiving immediate feedback. This dynamic exchange cultivates a culture of open dialogue and mutual learning.” (A6-PII)*

“*Depth and Meaning: Whether in-class or extracurricular, digital tools expand interactional diversity, enriching classroom atmospheres and deepening the intellectual significance of exchanges.” (A18-PII)*

### 4.4 Educational value of digital media technology on the “situation” and its systematic support for student mental health

Professors of drama education acknowledge that the digital media technology infrastructure currently provided by universities has significantly enhanced the effectiveness of drama education. In particular, digital media training laboratories focused on virtual technology offer a broader platform for teaching drama creation. Both teachers and students can utilize virtual reality technology to construct diverse theatrical settings and even create a wider range of dramatic characters. The digital media technology-enabled drama education environment exhibits characteristics of complexity, interconnectivity, and the integration of virtual and physical elements. By breaking through the spatial constraints of traditional drama education, digital media technology fosters a more multifaceted and developed educational setting. Furthermore, it extends the drama classroom infinitely, connecting it to a broader array of scenarios and resources. Additionally, the hybrid virtual–physical learning environment, primarily driven by virtual reality technology, provides greater imaginative possibilities for drama education.

#### 4.4.1 Transformations in educational environments

Drama educators argue that digital media technology facilitates the construction of multilayered educational ecosystems—spanning school, classroom, family, and societal environments—thereby fostering a conducive learning atmosphere for drama education. Digital media enhances both in-class and extracurricular dynamics while promoting the socialization of theater practices. An increasing number of institutions leverage technologies such as virtual reality (VR) to create immersive drama education spaces, enabling students to engage deeply with theatrical artistry. In classrooms, digital tools dismantle spatial constraints, enabling students to virtually connect with theaters and live performances and thereby bridge theory and practice. Beyond formal settings, digital media extends drama education into homes and broader societal contexts through resource-sharing platforms, democratizing access to learning opportunities.

“*Digital media also narrows the gap between educational institutions and external stakeholders, including theaters, industry partners, and government bodies. Remote connectivity, for instance, enables students to acquire real-world theatrical knowledge from professionals. Simultaneously, technological advancements empower students to participate in societal theater development—such as sharing creative works or critiques on social media platforms.” (A3-PII)*

“*Many institutions have established simulated drama studios equipped with digital tools, revolutionizing pedagogical environments. These studios provide students with hands-on opportunities to experiment and create, redefining experiential learning in drama education.” (A20-PII)*

In summary, digital media technology reconfigures educational environments—classrooms, schools, families, and society—by expanding the boundaries of drama education. This transformation not only enriches learning atmospheres but also integrates students into societal theater practices, accelerating the socialization and democratization of drama education.

#### 4.4.2 The systemic support of digital media technology for the mental health of drama education students

Professors of drama education assert that digital technology has disrupted the traditional, centralized, and standardized distribution model for drama education resources, establishing a personalized resource environment composed of cloud-based libraries and similar platforms. Through intelligent platforms, students can access drama materials tailored to their psychological characteristics and emotional states. Within such personalized learning environments, drama education students exhibit notable improvements in both learning engagement and self-efficacy. Consequently, drama education has evolved into a genuinely supportive and developmental space for psychological growth. Despite its benefits, digital technology may also have negative effects on students' mental health when overused. Prolonged screen time, constant online engagement, and reduced face-to-face interaction may contribute to mental fatigue, emotional strain, and a reduced sense of social connection. This suggests that the value of digital drama education depends on maintaining a balanced use of technology.

*The rich educational resources brought by digital technology allow students to select materials of different themes and levels of difficulty based on their own interests and needs through the school's cloud resource library. In some cases, the system even recommends suitable learning resources according to students' needs. This kind of personalized matching makes students feel that drama education is truly tailored to their needs, resulting in a significant improvement in their learning engagement and self-confidence. (A5-PII)*

## 5 Discussion

### 5.1 Digital media technology profoundly reshapes the role, positioning, and cognitive structure of drama education subjects

This study finds that the integration of digital media technology has led to a significant transformation in the role of drama education teachers, shifting them from traditional performance instructors to technology instructors who must acquire additional skills such as virtual character design and digital stage technology. Although some of their basic functions (e.g., knowledge explanation, assignment checking) have been replaced by relevant digital media technologies, the core of emotional expression in drama education remains reliant on teacher guidance. This finding is consistent with the view of Tian et al. (2023) in their study on teacher competency reconstruction as a key pillar of digital transformation in education, which argues that new technologies have given rise to an entirely new intelligent educational environment, where traditional knowledge instruction can be fully undertaken by intelligent robots, while teachers need to take on tasks that robots cannot perform, including facilitating interpersonal collaboration and guiding student development.

However, this does not imply that teachers simply become technology guides. Education in the intelligent era requires teachers to possess intelligent educational literacy that encompasses three dimensions—technology, education, and society (Zhang and Zhang, 2024). The use of digital technologies in higher education spaces introduces multiple risks, including dehumanization, spiritual degradation, loss of cognitive competencies, technologization and robotization, and threats to individual creative development (Kotlyarova et al., 2021). Therefore, in drama education, teachers must not only guide students in mastering technology but also lead them in mitigating the risks it poses, infusing greater humanistic care into the field.

Concurrently, this study reveals that the application of digital technology in drama education not only enhances students' artistic literacy and professional skills but also positively impacts their mental health. Positive attitudes toward digital technology and significantly improved digital literacy can enhance students' self-efficacy (Getenet et al., 2024). Digital media technology comprehensively enriches students' emotional experiences in drama education. Drama is inherently an art form that emphasizes emotional expression, emotional communication, and emotional identification. With the development of virtual reality technology, students in drama education can vividly convey emotional and value-laden content, enabling digital media technology to provide greater emotional value to otherwise abstract knowledge, thereby fostering greater emotional resonance and understanding in education.

Nevertheless, integrating digital technology into drama education inevitably introduces new psychological challenges. Although the use of personal computing technology has profoundly altered the landscape of interpersonal connectivity among college students, it appears to carry certain risks to mental health (Lattie et al., 2019). Shyroka et al. (2023), in their study on dehumanization in the digital educational process, argue that digital education displaces living teachers and students from the educational process, replacing them with technological means. This process erodes core educational elements such as critical thinking, emotional communication, and social capital, reducing education from “human cultivation” to “information transmission and skills training.” Therefore, in drama education, it is essential to balance technological dependence with autonomy, strengthen innovative capabilities with technological assistance, and avoid excessive immersion in instrumental applications.

### 5.2 Digital media technology drives systematic innovation in the forms and carriers of drama education content

This study finds that digital media technology has introduced new content dimensions into drama education, not only reshaping the values, ideas, and ways of thinking of teachers and students in university drama education but also stimulating their creativity. Internalizing digital media technology as an integral component of drama education is an inevitable choice in response to social development. Only by correctly recognizing its intrinsic value and proactively integrating it into the content system of drama education can we promote the sustainable development of drama education amid contemporary transformations, thereby meeting the objective of cultivating interdisciplinary drama talents. This finding echoes the conclusions of Zakopoulos et al. (2023), who found that the application of digital technology in drama education enhances students' awareness of sustainable development.

Technology, through the provision of rich digital resources, interactive platforms, and personalized learning pathways, has facilitated a paradigm shift from teacher-centered to student-centered instruction, significantly enhancing students' learning engagement, critical thinking, problem-solving skills, and digital literacy, essential 21st-century competencies (Kalyani, 2024). Technologies such as VR, AR, and MR have expanded the boundaries of dramatic expression. At the level of content carriers, traditional text and images have been transformed into immersive and interactive formats, significantly enhancing the authenticity of teaching and learner engagement. Big data and artificial intelligence technologies have enabled the personalized delivery of content, precisely matching student needs. At a deeper level, digital technology itself has become an inherent component of drama education, and an integrated “technology + arts” mindset should be progressively incorporated into the talent cultivation objectives of higher education institutions.

In addition, digital media technology, through functions such as multi-sensory narratives, intelligent emotional interaction, and cross-temporal and cross-cultural dialogue, has significantly expanded the emotional depth and psychological reach of the drama experience, constructing for students a space for psychological development characterized by emotional safety, freedom of expression, and a richness of meaning. By activating content, empowering processes, and deepening experiences, digital media technology enables students to release emotions through artistic expression, build confidence through virtual practice, and integrate the self through empathetic narratives, thereby forming a virtuous cycle in which artistic literacy and mental health develop synergistically. In the future, drama education should more consciously leverage the psychological nurturing function of digital technology, systematically integrating a mental health promotion perspective into curriculum design and instructional implementation, so that drama truly becomes a “theater of the soul.”

### 5.3 Digital technology reconstructs the interaction modes and behavioral characteristics of drama education

This study reveals that digital media technology has profoundly transformed the modes of interaction, relational structures, and participation mechanisms in drama education, giving rise to a novel instructional field characterized by multidimensional “human–technology–context” interactions. In drama education, teacher–student interactions leverage online discussions and intelligent feedback systems to transcend the limitations of time and space, enhancing efficiency and precision. Peer interactions, facilitated by social media and collaborative tools, stimulate cross-spatial cooperation and democratic expression. In human–computer interactions, intelligent robots and AI assistants serve as “technological partners,” enabling intelligent, context–aware interactions. This finding aligns with the research of Cadima et al. (2010) on the impact of learning technologies on instructional interaction design, which found that, compared to traditional static lectures, technology integration significantly enhances student engagement in classroom interactions, transforming learners from passive recipients into active participants, and fostering the development of critical thinking, creativity, communication, leadership, and teamwork skills.

School-based interventions that combine technology with effective pedagogy can promote positive social and mental health outcomes among students (Robinson and Van Ryzin, 2026). The digital transformation of the interaction process in drama education optimizes students' drama learning experiences across three dimensions: participation, immediacy, and support, while providing structural support for psychological development goals such as emotional management, social competence, and self-integration. The technology-facilitated interactive scenarios in drama education break the spatial and temporal limitations of traditional drama interactions, enabling students to engage in high-frequency, high-quality emotional expression training under lower psychological pressure. Drama education thus shifts from “performance for others” to “exploration of the self,” achieving deeper emotional awakening and psychological immersion through digital interaction.

Although the use of technological tools requires an initial adaptation phase, long-term practice can significantly enhance teachers' and students' technological proficiency and creativity, forming a positive cycle of “tool empowerment, cognitive upgrading, behavioral iteration.” By reshaping the interactive scenarios, feedback mechanisms, and relational structures of drama education, digital media technology transforms drama instruction from “skill transmission” into a multidimensional growth experience that integrates the psychological, social, and artistic dimensions. In the future, drama education should more consciously seize the opportunity presented by the digital transformation of interaction processes to construct “smart drama classrooms” that embody both the value of artistic education and the function of mental health promotion, making interaction itself an important vehicle for healing and growth.

### 5.4 Digital media technology reconstructs the learning ecology of drama education

This study finds that digital media technology constructs an immersive, intelligent, supportive, and open modern drama learning environment by systematically reshaping the physical space, resource ecology, emotional atmosphere, and cultural field of drama education. This finding aligns with the perspective of Fayez et al. (2021), who argued that smart learning can create a creative and comfortable learning environment, enabling learners to freely generate new ideas, interact, share, perceive, diagnose, and analyze their own learning processes. In terms of the physical environment, facilities such as virtual training rooms and 5G smart theaters support the integration of virtual and real elements in creation and rehearsal, enabling real-time connections with theaters and social resources through remote networking. The development of online platforms supports the educational process by enhancing students' communication, participation, collaboration, and motivation (Collazos et al., 2021). At the social level, families and communities are integrated into drama education through digital platforms (e.g., online resource sharing), promoting ubiquitous learning. Students engage in social drama practices through social media, thereby accelerating their professional socialization.

Metaverse technology can support various aspects of online classrooms, including realistic experiences, personalized teaching models, authentic 3D identities, interactive communication, virtual reality (VR) technology, and gamified learning. However, it also introduces several challenges, such as technical development difficulties, interaction issues, gaming addiction, privacy concerns, and ethical problems (Chen, 2024). Technology also has a dual effect: while national digital policies and industry talent gaps provide developmental dividends for education, issues such as rapid technological iteration, insufficient digital literacy among teachers, and the growing pains associated with the transformation of traditional theaters require systematic responses. Furthermore, Spytska (2025), reported that excessive use of digital technology may generate several adverse mental health outcomes, including cognitive decline, sleep disruption, and reduced physical activity. This observation is consistent with the interview data in the present study, in which some professors highlighted the harmful psychological consequences of overreliance on technology in education. Specifically, participants suggested that constant digital engagement, prolonged screen time, and persistent online academic demands may heighten stress, mental fatigue, and emotional strain among students. These findings suggest that while technology facilitates research, communication, and academic productivity, its excessive use may simultaneously jeopardize students' mental wellbeing.

Information overload, cyberbullying, and privacy anxiety within the technological environment also constitute new sources of psychological stress. At a deeper level, if the technology-mediated learning environment lacks genuine interpersonal interaction and emotional support, it may lead to a risk of dehumanization. Therefore, the future of drama education lies in transforming technology from a potential risk into an empowering vehicle for mental health, more consciously integrating environmental construction into the overall framework of mental health promotion. Through the systematic design of a “smart drama environment,” it could be possible to achieve an organic integration of artistic education space and psychological growth space, enabling the environment itself to serve as a silent educator and a gentle guardian.

## 6 Conclusion

This study employs an ethnographic research method, utilizing in-depth interviews with drama education professors, to systematically explore the value of digital media technology in drama education and its impact on students' mental health. The findings reveal that digital media technology profoundly drives the digital transformation of drama education by reshaping teacher–student roles, reconstructing educational content, innovating interaction forms, and expanding the learning environment, thereby demonstrating multidimensional educational value. At the “Subject” level, teachers extend from traditional “performance instructors” to “technology instructors,” yet the emotional core of drama education still requires teacher guidance, with technology only replacing basic functions such as knowledge transmission. Meanwhile, students gain richer pathways for self-expression and psychological growth through technological empowerment. At the “content” level, digital technology not only expands the knowledge boundaries of drama education but also significantly enhances students' cognitive engagement and emotional experiences through immersive presentation and personalized delivery.

At the “behavior” level, technology reconstructs the interaction modes between teachers and students, among peers, and between humans and machines, transforming drama instruction from “skill transmission” into a multidimensional growth experience that integrates psychological, social, and artistic dimensions. At the “situation” level, digital technology constructs an immersive, intelligent, and open ubiquitous learning ecosystem, providing a more supportive physical and psychological space for drama education. By offering new emotional support, actively regulating, and synergistically promoting students' mental health, digital media technology in drama education can effectively enhance students' psychological resilience and social adaptability, providing a feasible pathway for achieving the dual objectives of improving teaching quality and empowering psychological development in the new era of education.

Meanwhile, this study also reveals the risks and challenges associated with the application of digital technology. Excessive reliance on technology may lead to a crisis of dehumanization, eroding core educational elements such as critical thinking, emotional communication, and social capital. Issues such as information overload, cyberbullying, and technology addiction may also become new sources of psychological stress. Therefore, the integration of digital technology into drama education should not be understood as a simple substitution of technology for human presence, but rather as a symbiotic integration of technology and art. The key lies in being guided by clear educational goals, supported by positive interpersonal interaction, and emphasizing structure and moderation, so that technology can be transformed from a potential risk into an empowering vehicle for mental health. In the future, drama education must remain “human-centered,” attending to students' mental health while balancing technological innovation, artistic essence, and psychological development, cultivating a new generation of drama talents who possess digital literacy, creativity, and social responsibility. Only in this way can technology truly serve the holistic development of individuals and sustain the vitality of the inheritance and innovation of the art of drama.

Finally, this study offers important insights from the perspectives of drama professors and teachers, it does not directly capture students' own perspectives on the issues examined. Future studies are encouraged to include students' voices and experiences in order to deepen international academic discussion and to develop a more comprehensive and balanced understanding of this field.

## Data Availability

The raw data supporting the conclusions of this article will be made available by the authors, without undue reservation.
